# Involvement of gelsolin in TGF-beta 1 induced epithelial to mesenchymal transition in breast cancer cells

**DOI:** 10.1186/s12929-015-0197-0

**Published:** 2015-10-20

**Authors:** Zhi-Yuan Chen, Pei-Wen Wang, Dar-Bin Shieh, Kuan-Ying Chiu, Ying-Ming Liou

**Affiliations:** Department of Life Sciences, National Chung-Hsing University, Taichung, 40227 Taiwan; Institute of Basic Medical Sciences, National Cheng Kung University, Tainan, 701 Taiwan; Institute of Oral Medicine, National Cheng Kung University, Tainan, 701 Taiwan; Rong Hsing Research Center for Translational Medicine, National Chung Hsing University, Taichung, 40227 Taiwan

**Keywords:** GSN, TGF-β1, EMT, Methylation specific PCR, DNA methyltransferases

## Abstract

**Background:**

Increasing evidence suggests that transforming growth factor-beta 1 (TGF-β1) triggers epithelial to mesenchymal transition (EMT) and facilitates breast cancer stem cell differentiation. Gelsolin (GSN) is a ubiquitous actin filament-severing protein. However, the relationship between the expression level of GSN and the TGF-β signaling for EMT progression in breast cancer cells is not clear.

**Results:**

TGF-β1 acted on MDA-MB231 breast cancer cells by decreasing cell proliferation, changing cell morphology to a fibroblast-like shape, increasing expressions for CD44 and GSN, and increasing EMT expression and cell migration/invasion. Study with GSN overexpression (GSN op) in both MDA-MB231 and MCF-7 cells demonstrated that increased GSN expression resulted in alterations of cell proliferation and cell cycle progression, modification of the actin filament assembly associated with altering cell surface elasticity and cell detachment in these breast cancer cells. In addition, increased cell migration was found in GSN op MDA-MB231 cells. Studies with GSN op and silencing by small interfering RNA verified that GSN could modulate the expression of vimentin. Sorted by flow cytometry, TGF-β1 increased subpopulation of CD44+/CD22- cells increasing their expressions for GSN, Nanog, Sox2, Oct4, N-cadherin, and vimentin but decreasing the E-cadherin expression. Methylation specific PCR analysis revealed that TGF-β1 decreased 50 % methylation but increased 3-fold unmethylation on the GSN promoter in CD44+/CD22- cells. Two DNA methyltransferases, DNMT1and DNMT3B were also inhibited by TGF-β1.

**Conclusions:**

TGF-β1 induced epigenetic modification of GSN could alter the EMT process in breast cancer cells.

## Background

TGF-β1 is a secreted cytokine involved in controlling gene expression and ultimately cell cycle and tissue repair [1]. In the initial stage of tumorigenesis, TGF-β1 acts as a tumor suppressor [2]. With tumor progression cancer cells overproducing TGF-β1 turn to promote cancer cell proliferation, invasion and metastasis, hence become resistant to the TGF-β1-induced growth inhibition in their later stage [2]. In addition, the TGF-β1 signaling pathway has been shown to cause a constitutive epithelial to mesenchymal transition (EMT) facilitating a highly invasive and metastatic phenotype in breast tumors [3, 4]. Recent evidence also demonstrated that TGF-β could increase breast tumor-initiating cell numbers in the low claudin expression subtype of breast tumors [5]. Apparently, the TGF-β signaling for EMT, cell motility, and invasiveness might play an important role in enriching the cancer stem cell (CSC) pool in breast tumors [6].

The actin cytoskeleton underlies several cellular functions including cell differentiation in both normal and tumor cells [7–10]. It has been shown that the TGF-β signaling via Smad and p38MAPK caused upregulation of actin binding proteins, including tropomyosin, α-actinin, and calponin, to control the stress fiber formation, which might contribute to modulation of cell motility and invasive phenotype with EMT in tumor cells [11, 12]. Gelsolin (GSN), one of the most potent members of the actin-severing superfamily, plays a key role in the regulation of actin filament assembly and disassembly [13, 14]. GSN involves in many cellular properties for carcinogenesis phenotypes, EMT, motility, apoptosis, proliferation, and differentiation [15–17]. However, it remains to be determined if the TGF-β signaling events also include the modulation of GSN expression for promotion of breast cancer cell differentiation.

Similar to leukemia, several CSC-like subpopulations have been thought to exist in breast cancers [18, 19]. These breast CSCs acquire the ability to differentiate into all the different cells found within a tumor that become chemotherapy resistant [20, 21]. In this study, the TGF-β1-induced MDA-MB231breast cancer cells as a model for CSC differentiation were used to investigate whether the expression level of GSN is regulated by the TGF-β1 signaling for promoting breast CSC differentiation. Results reported here suggest that GSN involves in the TGF-β1-driving CSC differentiation by the process of EMT in breast cancer cells.

## Methods

### Cell culture and TGF-β1 treatment

MCF-7 cells in Dulbecco’s modified essential medium (DMEM, Gibco) containing glutamine, while MDA-MB231 in DMEM containing sodium bicarbonate, both supplemented with antibiotics and 10 % fetal bovine serum (FBS) were cultured in a 5 % CO_2_ incubator at 37 °C. To determine the growth inhibition, 5000 cells for each cell line were plated in 96-well plates with or without TGF-β1 treatment. Cell viability and proliferation was measured using the MTT [3-(4,5-dimethylthiazol-2-yl)-2,5-diphenyltetrazolium bromide] assay (ATCC, Manassas, VA, USA) [22, 23].

### Cell migration and invasion assay

The invasion and migration assay of cancer cells were performed using modified Boyden chamber assay with a *FalconTM Cell Culture Insert* (BD Biosciences). To create an invasion assay the membrane was coated with a Matrigel to simulate the typical matrices that cancer cells encounter during the invasion process in vivo. In contrast, the membrane without coating was used only for the migration assay. In both measurements, the cells (10^5^ cells/ml) were placed on upper side and a chemoattractant (10 % FBS) on the lower side. Cells that migrated through the membrane were fixed with 100 % absolute alcohol, stained with crystal violet. After air dried, migrated cells were then added with 30 % acetic acid, and quantitated by measuring the optical density at 590 nm in a micro-plate reader.

### Cell cycle phase determination

Cells (10^7^) were seeded in a 10-cm dish in DMEM-0.2 % FBS and cultured in a CO_2_ incubator at 37 °C for 24 h. The cells were then changed to fresh medium, trypsinized, and centrifuged. The pellet was washed and re-suspended in 1 ml of pre-chilled phosphate buffer solution (PBS) and the cells fixed by gradually adding 3 ml of 95 % ethanol, then were stored in a deep freezer (−20 °C) overnight. The cells were then washed three times by centrifugation and resuspension in pre-chilled PBS. To stain the cells with propidium iodide (PI), the cells were resuspended in PBS containing 0.1 % Triton X-100, 20 μg/ml of PI, and 0.2 mg/ml of RNase A and incubated for 30 min at room temperature in the dark. Samples were analyzed on a flow cytometer (FC500 Flow Cytometry System, Beckman Coulter, Inc.) with a 488 nm excitation laser. The cell cycle phases were determined using the computerized software provided with the machine (CXP Software, Beckman Coulter, Inc.).

### Cell staining for FACS flow cytometry

Cells (10^7^) were incubated with fluorochrome-conjugated antibodies followed by fluorescence-activated cell sorting (FACS). To characterize stem cell markers in breast cancer cells, the following antibodies were used: allophycocyanin (APC)-conjugated anti-human CD44 (clone G44-26, mouse IgG2b, BD Pharmingen, CA, USA), phycoerythrin (PE)-conjugated anti-human CD24 (clone ML5, mouse IgG2a, BD Pharmingen, CA, USA). Single-cell suspensions dissociated from the dishes, using cell dissociation buffer (GIBCO), were stained with flurochrome antibody for 30 min at 4 °C and analyzed by a flow cytometry of BD FACS Aria apparatus (BD Biosciences, Palo Alto, CA).

### RNA extraction, semi-quantitative RT-PCR, real-time qPCR, comparative CT method for quantification of mRNA expression

The procedures for RNA extraction, semi-quantitative reverse transcription polymerization chain reaction (semi-quantitative RT-PCR), and qPCR were described previously [22–24]. SYBR Green dye was used as a real-time reporter of the presence of double-stranded DNA. The following primers specific for stem cell markers (i.e. Oct4, Sox2 and Nanog), for EMT markers (i.e. N-cadherin, and vimentin, and E-cadherin), for GSN, and for DNMT1/DNMT3B were synthesized: Oct4, forward 5′-CCTGAAGCAGAAGAGGATCA-3′ and reverse 5′-CCGCAGCTTACACATGTTCT-3′; Sox2, forward 5′-CGATGCCGACAAGAAAACTT -3′ and reverse 5′-CAAACTTCCTGCAAAGCTCC-3′; Nanog, forward 5′-TTCAGTCTGGACACTGGCTG-3′ and reverse 5′-CTCGCTGATTAGGCTCCAAC-3′; E-cadherin, forward 5′-GCCTCCTGAAAAGAGAGTGGAAG-3′ and reverse 5′-TGGCAGTGTCTCTCCAAATCCG-3′; N-cadherin, forward 5′-ACAGTGGCCACCTACAAAGG-3′ and reverse 5′-CCGAGATGGGGTTGATAATG-3′; Vimentin, forward 5′-AGGAAATGGCTCGTCACCTTCGTGAATA-3′ and reverse 5′-GGAGTGTCGGTTGTTAAGAACTAGAGCT-3′; GSN, forward 5′-ACGGACCCAGCCAATCG-3′ and reverse 5′-CATCATCCCAGCCAAGGAA-3′; DNMT1, forward 5′-AAGACAAAGACCAGGATGAGAAG-3′ and reverse 5′-GGGTGTTGGTTCTTTGGTTTG-3′; DNMT3B, forward 5′-CCATTCGAGTCCTGTCATTG-3′ and reverse 5′-GCAATGGACTCCTCACACAC-3′. The primers for actin binding proteins were: Tropomyosin 1 (Tm1), forward 5′-TCATCATTGAGAGCGACCTG-3′ and reverse 5′-CTTGTCGGAAAGGACCTTGA-3′; Caldesmon, forward 5′-CTGGCTTGAAGGTAGGGGTTT -3′ and reverse 5′-TTGGGAGCAGGTGACTTGTTT-3′; Profilin, forward 5′-CTGTCAGGACGCGGCCATCG-3′ and reverse 5′-AACGTTTTCCCGGGGACGGC-3′. GAPDH, an internal control, had the forward primer 5′-ATGGGGAAGGTGAAGGTCG-3′ and the reverse primer 5′-TAAAAGCAGCCCTGGTGACC-3′, respectively.

### Immunoblotting

Protein contents of total cell lysates from TGF-β1 treated or untreated cells were analyzed by western blot. Samples with same amounts of protein were separated by sodium dodecyl sulfate-polyacrylamide gel electrophoresis, then the proteins were electro-transferred onto polyvinylidene difluoride membranes. The primary antibodies used were: mouse monoclonal anti-human GSN (Sigma GS-2C4; 1:10000 dilution), mouse monoclonal anti-human CD44 (Abcam;1:1000 dilution), mouse monoclonal anti-human E-cadherin (2Q663) (sc-71008), human-β-catenin (9 F2) (sc-47752), human GSK-3β (H-76) (sc-9166), human cyclin D1 (DSC-6) (sc-20044), mouse monoclonal anti-human N-cadherin (H-63) (sc-7939) (all from Santa Cruz; 1: 2000 dilution), mouse monoclonal anti-β-actin (Sigma, 1: 10000 dilution), rabbit monoclonal anti-Tm1 (Sigma, 1:2000 dilution), rabbit monoclonal anti-caldesmon (Santa Cruz, 1:5000 dilution), rabbit monoclonal anti-profilin (Santa Cruz, 1:3000 dilution), rabbit monoclonal anti-human vimentin (Abcam; 1:1000 dilution), and rabbit polyclonal anti-GAPDH (GeneTex GTX100118; 1:5000). The secondary antibodies used (1:5000 dilution) were goat anti-rabbit IgG (Sigma), and goat anti-mouse IgG (Sigma).

### GSN overexpression and silencing by small interfering RNA (siRNA)

The pc6-GSN plasmid construct was cotransfected with GSN into both MCF-7 and MDA-MB231 breast cancer cells using lipofectamine 2000 (Invitrogen). The full-length cytoplasmic GSN cDNA [24, 25] was cloned into the expression vector pcDNA6-V5/His. Before transfection, cells were cultured in a 6-well plate containing culture medium without antibiotics at a density of 70-80 % confluence. Both the lipofectamine and DNA constructs were diluted with transfection medium without serum and incubated for 5 min. Subsequently, the diluted DNA constructs and diluted lipofectamine were mixed at a 1:2.5 ratio of DNA to lipofectamine. After gentle shaking and incubation for 20 min, the DNA-lipofectamine complexes were added to each well and incubated in a CO_2_ incubator at 37 °C for 6 h. The culture medium was replaced with serum-containing DMEM.

Sixty to eighty percent confluent cells were transfected with siRNAs directed to human GSN (sc-7330), according to the manufacturer’s guidelines (Santa Cruz Biotechnology). The cells received 10 μM siRNA were incubated for 6 h at 37 ° C in a CO_2_ incubator. At 24 and 48 h after transfection total RNA were extracted for reverse transcription and qPCR measurements to confirm downregulation of GSN expression [22, 24].

### Atomic force microscopy

DI-Dimension 3100 AFM (Digital Instruments, Santa Barbara, CA) was applied to obtain cell surface contour images in contact mode and measure the interfacial forces in tapping mode [24]. The V-shaped silicon cantilevers with a spring constant of ~0.9 to 0.12 N/m were used for imaging cell surface areas (20 × 20 μm) in phosphate buffer. Approximately 10–20 spots of this scanning region were randomly selected using the same probe to extend forward 1 nm deep and to retract back to the starting point. The retracting force-distance curves were used to calculate the adhesion forces that correspond to the elasticity of cell membrane surface.

### Cell detachment measurements

Cells (10^5^) plated on a 12-well plate were treated with 150 μl of TrypLETM (Invitrogen) for 60, 90, and 180 s, respectively, followed by adding 1 ml of cell culture medium to stop the action of trypsin. Detached cells post treatments with TrypLETM for 60, 90, and 180 s were collected and measured the cell number by trypan blue exclusion assay. The detached cell numbers for the group with 180 s post trypsin treatment were used to normalize the degree of cell detachment for the group with 60 or 90 s after trypsin treatments.

### Methylation-specific PCR analyses

Methylation status of GSN was determined by methylation-specific PCR (MSPCR) using bisulphate-modified genomic DNA as the template. Genomic DNA was treated with bisulphate by using the Zymo DNA Modification Kit (Zymo Rearch, Orange, CA, USA) according the protocol provided by the manufacturer. Methylation-specific GSN primers are: forward: 5’-ATGTTTATTTGATAAACGAGGGAAAC-3’, and reverse: 5’-CATTAAACAAACGCCTCGAA-3’; and unmethylation-specific GSN primers: forward: 5’-GTTTATTTGATAAATGAGGAAATGG-3’ and reverse: 5’-TAAACCATTAAACACCTCAAA-3’.

### Statistical analysis

Quantitative values are presented as the mean and standard error of the mean (mean ± SEM). A difference was considered to be statistically significant when the P value was less than 0.05.

## Results

### Effects of TGF-β1 treatment on cell proliferation, the expression of CD44, GSN, and EMT markers (i.e. N-cadherin, vimentin, and E-cadherin) in MDA-MB231 breast cancer cells

**Fig. 1:**
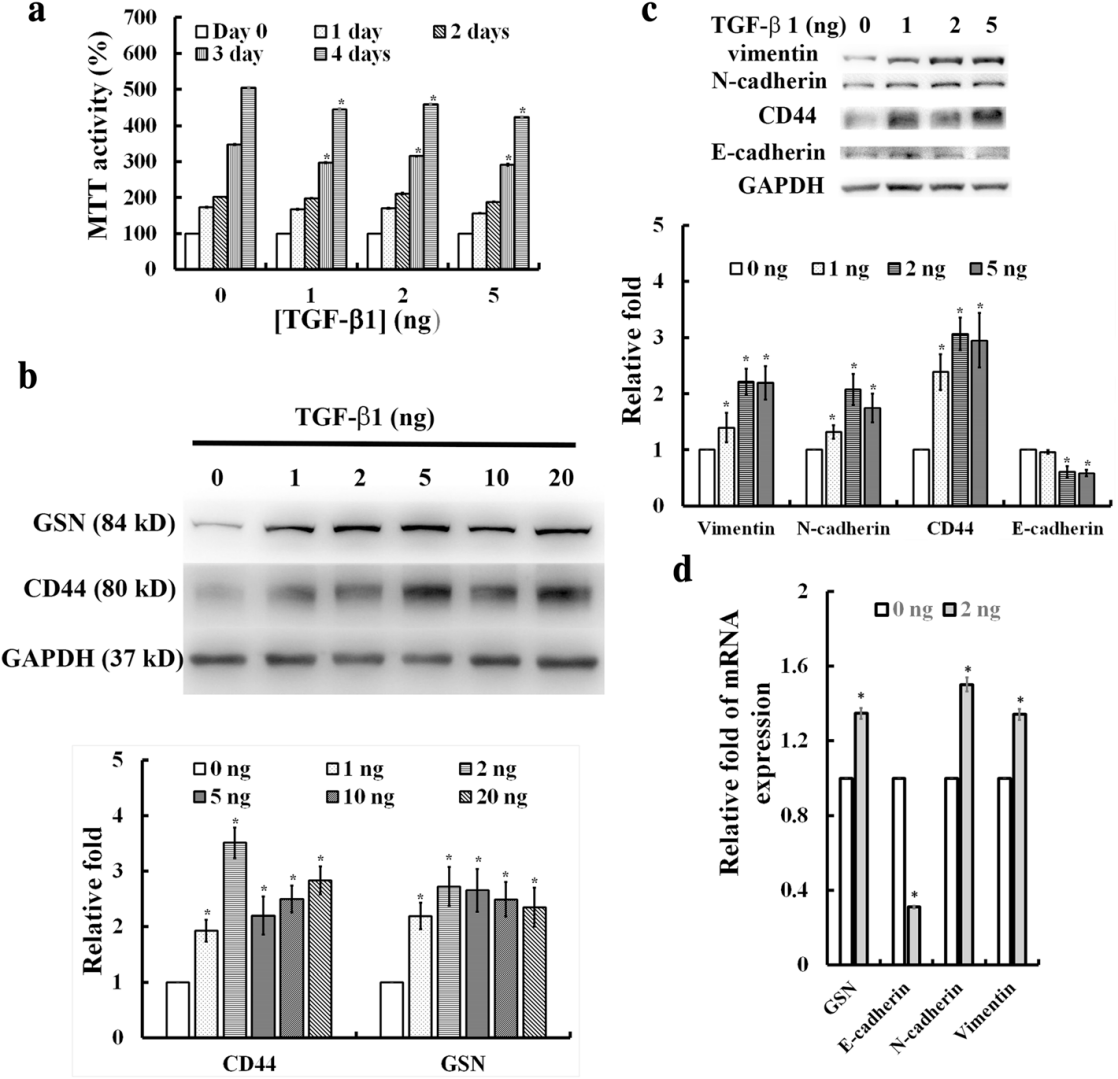
Effects of TGF-β1 treatment on cell proliferation, the expression of CD44, GSN, and EMT markers (i.e. N-cadherin, vimentin, and E-cadherin) in MDA-MB231 breast cancer cells. **a** MTT assay of cell viability after incubation of MDA-MB231 breast cancer cells with TGF-β1 from 1 to 5 ng/ml for 0, 24, 48, 72, and 96 h. **b** Western blotting (top) with quantitative analyses (bottom) showed a dose dependent increase in protein expressions for CD44 and GSN in MDA-MB231 cells treated with 0, 1, 2, 5, 10, 20 ng/ml of TGF-β1 for 72 h. **c** Western blotting (top) with quantitative analyses (bottom) of vimentin, N-cadherin, CD44, and E-cadherin levels in MDA-MB231cells treated with 0, 1, 2, 5 ng/ml of TGF-β1 for 72 h. GAPDH used as an internal control. **d** Real-time quantitative PCR (qPCR) analysis showed the mRNA level for GSN, E-cadherin, N-cadherin, and vimentin in MDA-MB231 cells treated with or without 2 ng/ml TGF-β1 for 3 days. In **a**, **b**, **c**, and **d**, the values are the mean ± SEM (*n* = 6), with * indicating a significant difference compared to the untreated cells

To test the appropriate condition for TGF-β1 induction, MDA-MB231 breast cancer cells were treated with TGF-β1 from 1 to 20 ng/ml for 0, 24, 48, 72, and 96 h. Treatment with > 1 ng/ml TGF-β1 for 72 h sufficiently decreased cell proliferation (Fig. 1a) in MDA-MB231 cells. Applying different concentrations (1, 2, 5, 10, 20 ng/ml) of TGF-β1 to cells in culture medium for 3 days, MDA-MB231 cells showed a dose dependent increase in protein expressions for CD44 and GSN (Fig. 1b). In addition, MDA-MB231 cells treated with TGF-β1 from 1 to 5 ng/ml for 3 days also increased the expression of mesenchymal cell markers (i. e. N-cadherin, vimentin) but decreased the expression for epithelial cell marker (i.e. E-cadherin) (Fig. 1c). In parallel, the mRNA level was increased for GSN and N-cadherin and vimentin but decreased for E-cadherin in MDA-MB231 cells treated with 2 ng/ml TGF-β1 for 3 days, as compared to control without TGF-β1 (Fig. 1d). In addition, the TGF-β1 treatment was confirmed to facilitate cell migration and invasion in MDA-MB231 breast cancer cells.

### Effects of GSN op on cell proliferation, cell cycle progression, and the expression of GSK-3β, β-catenine, and cyclin D1 in MDA-MB231 and MCF-7 breast cancer cells

**Fig. 2:**
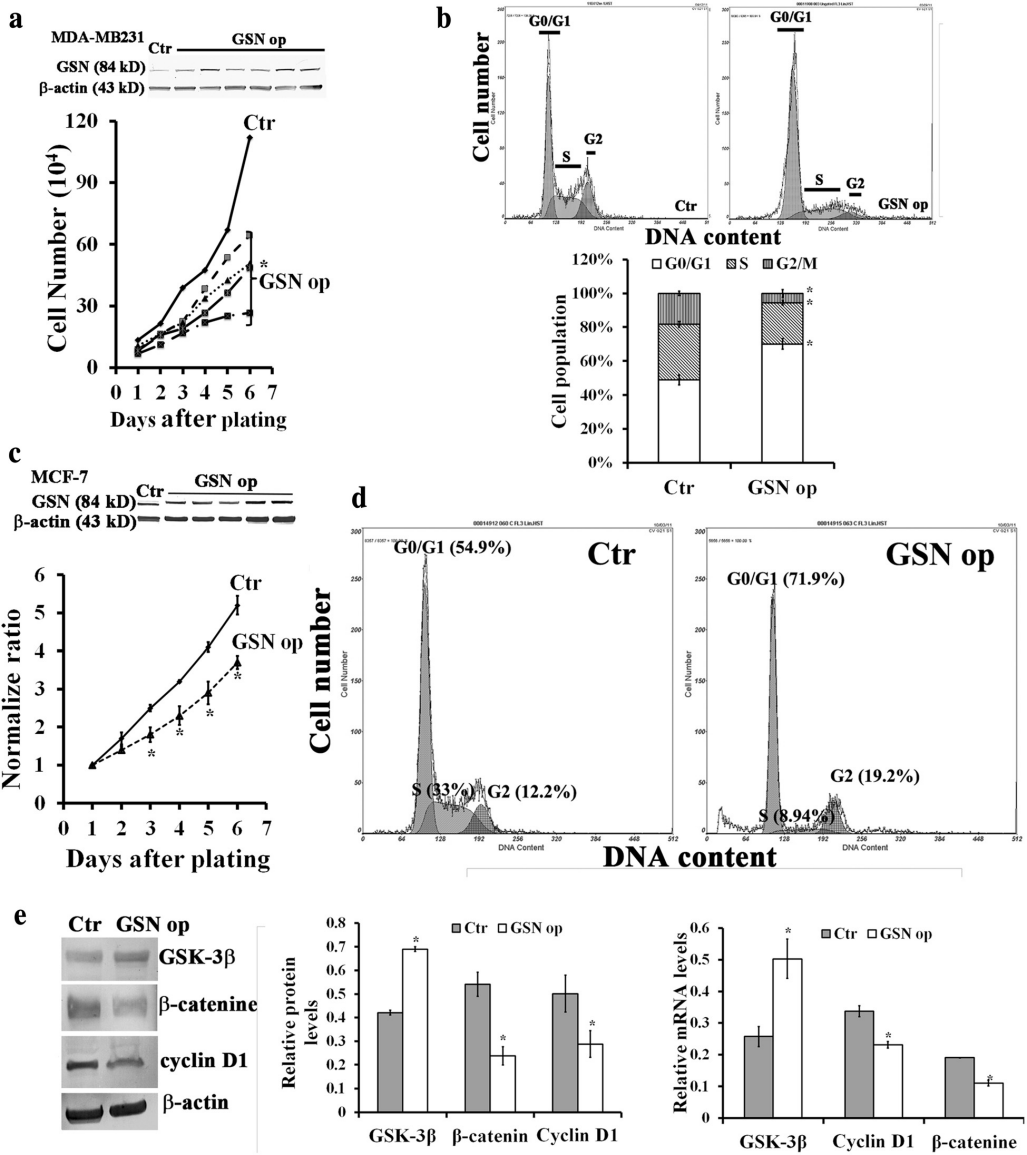
Effects of GSN op on cell proliferation, cell cycle progression, and the expression of GSK-3β, β-catenine, and cyclin D1 in MDA-MB231 and MCF-7 breast cancer cells. GSN overexpression increased the doubling time for cell proliferation in stable GSN-overexpressing (GSN op) clones of (**a**) MDA-MB231, and (**c**) MCF-7 breast cancer cells as compared to their controls (Ctr), respectively. Flow cytometry determined the cell population at different cell cycle phases: G0/G1, S, and G2 in control and GSN op cells of (**b**) MDA-MB231, and (**d**) MCF-7 cells, respectively. **e** GSN overexpression altered the protein levels and mRNA expression of GSK-3β, β-catenin, cyclin D1 in MDA-MB231 or MCF-7 cells. β-actin used as an internal control. In **a**, **b**, **c**, **d** and **e**, the values are the mean ± SEM (n = 6), with * indicating a significant difference compared to the cells in control and GSN op, respectively

To further determine the functional role of increased GSN expression in the TGF-β induced signaling for modulation of breast cancer cell progression, we conducted GSN overexpression (GSN op) in the two human breast cancer cell lines of MDA-MB231 and MCF-7. Stable clones of GSN op cells have 2- to 6- folds of GSN overexpression and the longer doubling time for cell proliferation in both MDA-MB231 and MCF-7 cells (Fig. 2a and c). This is consistent with the finding that TGF-β1 treatment decreased cell proliferation (Fig. 1a) with increased GSN expression (Fig. 1b and d) in MDA-MB231 cells. Flow cytometry also revealed that cell cycle progression arrest at G0/G1 phase accompanying by halting cell cycle progression to DNA synthesis (S phase) occurred in GSN op MDA-MB231 and GSN op MCF-7 cells as compared to their controls, respectively (Fig. 2b and d).

Glycogen synthase kinase-3β (GSK-3β) is a key component of multiple signaling pathways involved in the regulation of cell fate, protein synthesis, glycogen metabolism, cell mobility, proliferation, and survival [26–28]. By preventing cells from entering the cell cycle, GSK-3β participates in the regulation of the β-catenin signaling pathway by modulating cyclin D1 expression levels [29]. As compared to control cells, the expression levels of GSK-3β was also increased in concomitant with the decrease of the levels of cyclin D1 and β-catenin in GSN op MDA-MB231 and GSN op MCF-7 cells, which may cause the subsequent cell cycle arrest at the G1-S phase and hence halting DNA synthesis in those cells (Fig. 2e).

### Effects of GSN op on changes of cell morphology, cell surface elasticity, cytoskeletal protein expression, cell detachment, and migration in MDA-MB231 breast cancer cells

**Fig. 3:**
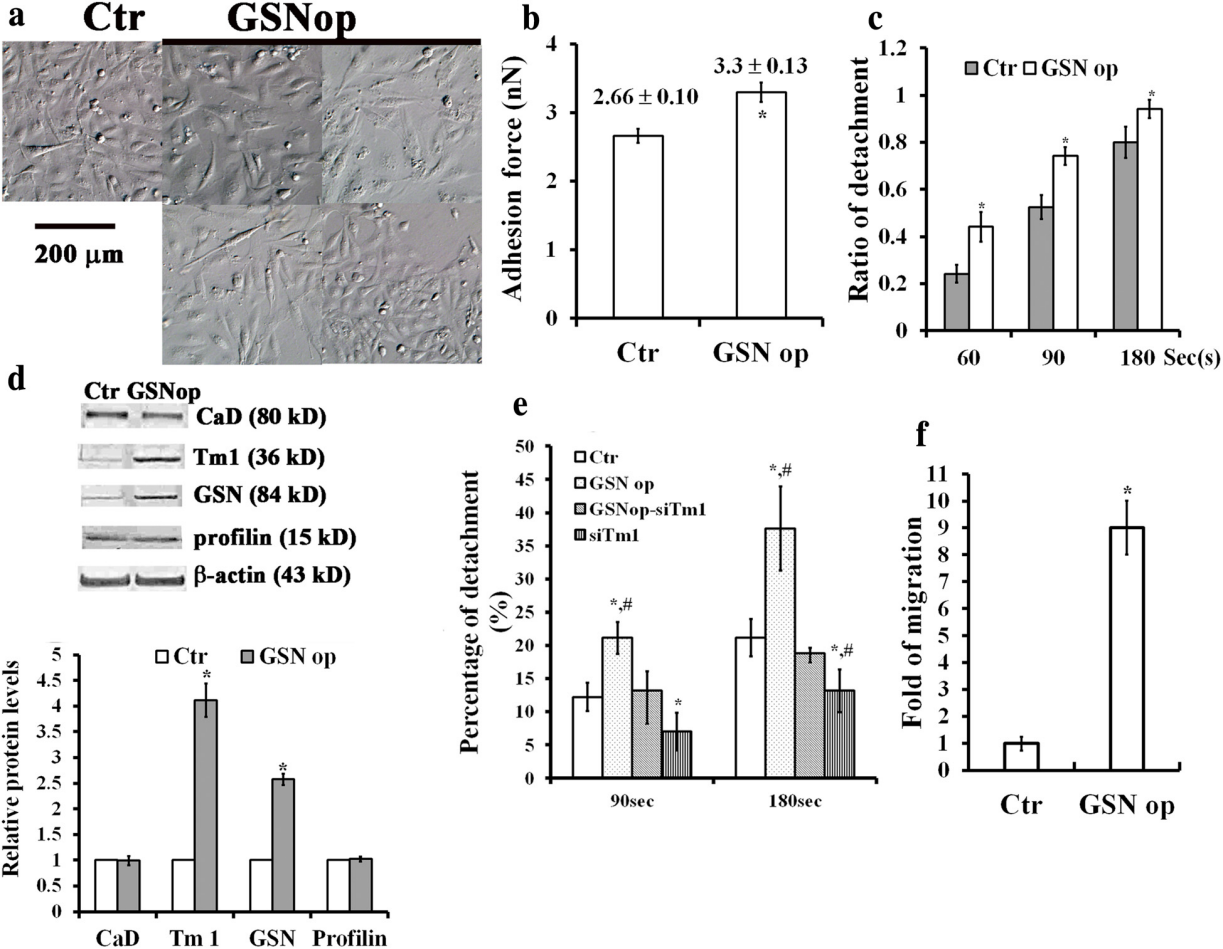
Effects of GSN op on changes of cell morphology, cell surface elasticity, cytoskeletal protein expression, cell detachment, and migration in MDA-MB231 breast cancer cells. **a** GSN op altered cell morphology and (**b**) increased cell surface elasticity in MDA-MB231 cells. Four different stable GSN transfected MDA-MB231 cell clones were used. The calibration bar is 200 μm. Atomic force microscopy was applied to obtain with quantitative analysis of the cell-surface adhesion force in control and GSN op MDA-MB231 cells. **c** Effect of GSN op on cell detachment for MCF-7 cells. **d** Effect of GSN op on the protein levels shown by immunoblotting (top) with quantitative analyses (bottom) for caldesmon (CaD), tropomyosin 1 (Tm 1), GSN and profilin (Pro) in MDA-MB231 cells. β-actin used as an internal control. **e** Effect of siTm 1 on cell detachment for MDA-MB231cells with or without GSN op. **f** Effect of GSN on the cell migration in MDA-MB 231 cells. In **b, c, d, e, f**, the values are the mean ± SEM (*n* = 40 in **b**, *n* = 6 in **c**, **d**, **e**, **f**), with *, # indicating a significant difference compared to the cells in control and GSNop-siTm, respectively

In comparison with control MDA-MB 231 cells (Ctr), several GSN op clones of MDA-MB231 cells were found to alter morphological changes in cell shapes (Fig. 3a) with increased cell surface elasticity (Fig. 3b). Cell surface elasticity was determined by measuring adhesion force in the control and GSN op MDA-MB 231 cells (Fig. 3b). The adhesion force measured on the cell surface was 2.66 ± 0.10 and 3.30 ± 0.13 nN for the control and GSN op cells, respectively (Fig. 3b). Clearly, the upregulation of GSN could alter the cell surface adhesion associated with morphological modification in breast cancer cells. Since the dynamic formation of cell surface adhesion and detachment is required for cancer cell motility and invasion [30], we also determined the effect of GSN op on the cell detachment in breast cancer cells (Fig. 3c). Result obtained showed that cell detachment from extracellular matrix was increased for GSN op cells as compared to control cells (Fig. 3c). This suggested that GSN severing the actin filament might contribute to offset the cell adhesion and or detachment to extra-cellular matrices in breast cancer cells. Interestingly, GSN op MDA-MB231 cells were also found to increase the protein content for Tropomyosin 1 (Tm1) as compared to controls (Fig. 3d). This is consistent with our previous finding that both GSN and Tm1 could affect the cell surface adhesion and cell proliferation in breast cancer cells [22]. To determine the effect of increased Tm1 expression levels on the cell detachment in GSN op MDA-MB231cells, siTm1 was conducted in the control and GSN op cells, respectively (Fig. 3e). Gene silencing Tm1 caused to decrease the cell detachment in both control and GSN op MDA-MB231 cells (Fig. 3e), suggesting that upregulation of Tm 1 by GSN op might contribute to facilitate cell detachment in MDA-MB231 cells. To verify that increases in GSN severing the actin filament caused to enhance cell motility in breast cancer cells, cell migration assay was compared in control and GSN op MDA-MB 231 cells (Fig. 3f). The result showed that GSN op significantly enhanced cell migration (~9 fold) in MDA-MB231 cells (Fig. 3e). This is consistent with the previous finding that down-regulation of GSN family proteins in MDA-MB 231 cells reduced the invasive and motile properties of breast cancer cells [31].

### Effects of GSN op and/or silencing by small interfering RNA on the expression of vimentin

**Fig. 4:**
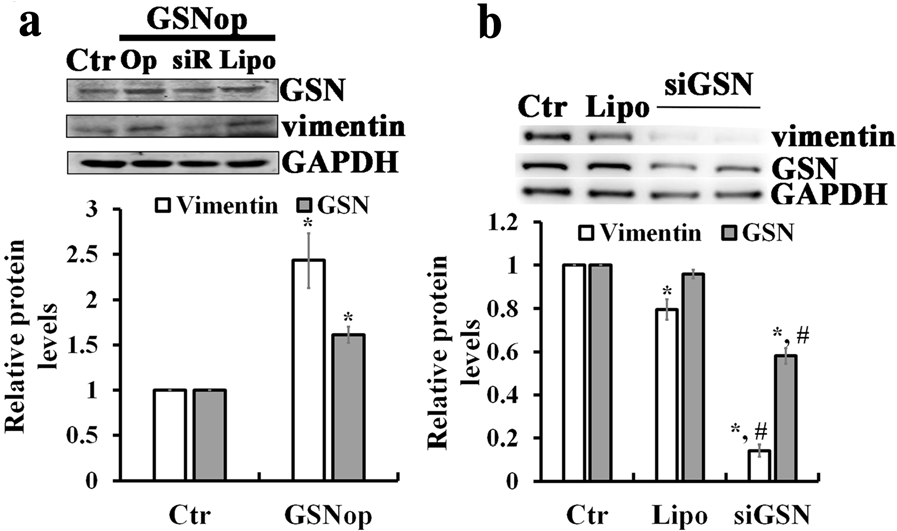
Effects of GSN op and/or silencing by small interfering RNA on the expression of vimentin in MDA-MB231 breast cancer cells. **a** Western blotting (top) with quantitative analyses (bottom) of GSN, and vimentin, levels in control MDA-MB231 cells (Ctr), and GSN op MDA-MB231cells treated with and/ or without siRNA against GSN (siR) or lipofemitamine (Lipo). **b** Effect of siGSN on the protein content of vimentin, and GSN in MDA-MB231 cells (Ctr). GAPDH used as an internal control. In **a** and **b**, the values are the mean ± SEM (n = 3), with *, # indicating a significant difference compared to the control cells and Lipo-treated cells, respectively

To confirm that GSN plays a crucial role in the TGF-β1 induced EMT in breast cancer cells, studies with GSN op and/or siGSN were conducted in MDA-MB231 cells for measuring their effects on the expression levels of mesenchymal cell marker, vimentin (Fig. 4). Western analysis showed that in GSN op MDA-MB231 cells GSN op significantly increased the protein content of vimentin as compared to control cells without GSN op (Fig. 4a). The increase in the protein content of GSN and vimentin returned to the control level with siRNA treatment on GSN op cells (Top panel of Fig. 4a). In contrast, MDA-MB231 cells treated with siGSN caused decreases in the protein contents of vimentin and GSN by ~86 % and ~42 %, respectively (Fig. 4b).

### TGF-β1 induction increases the CD44+/CD24- subpopulation by coordinating gene expressions for CSC markers, EMT markers, and GSN in MDA-MB231 cells

**Fig. 5:**
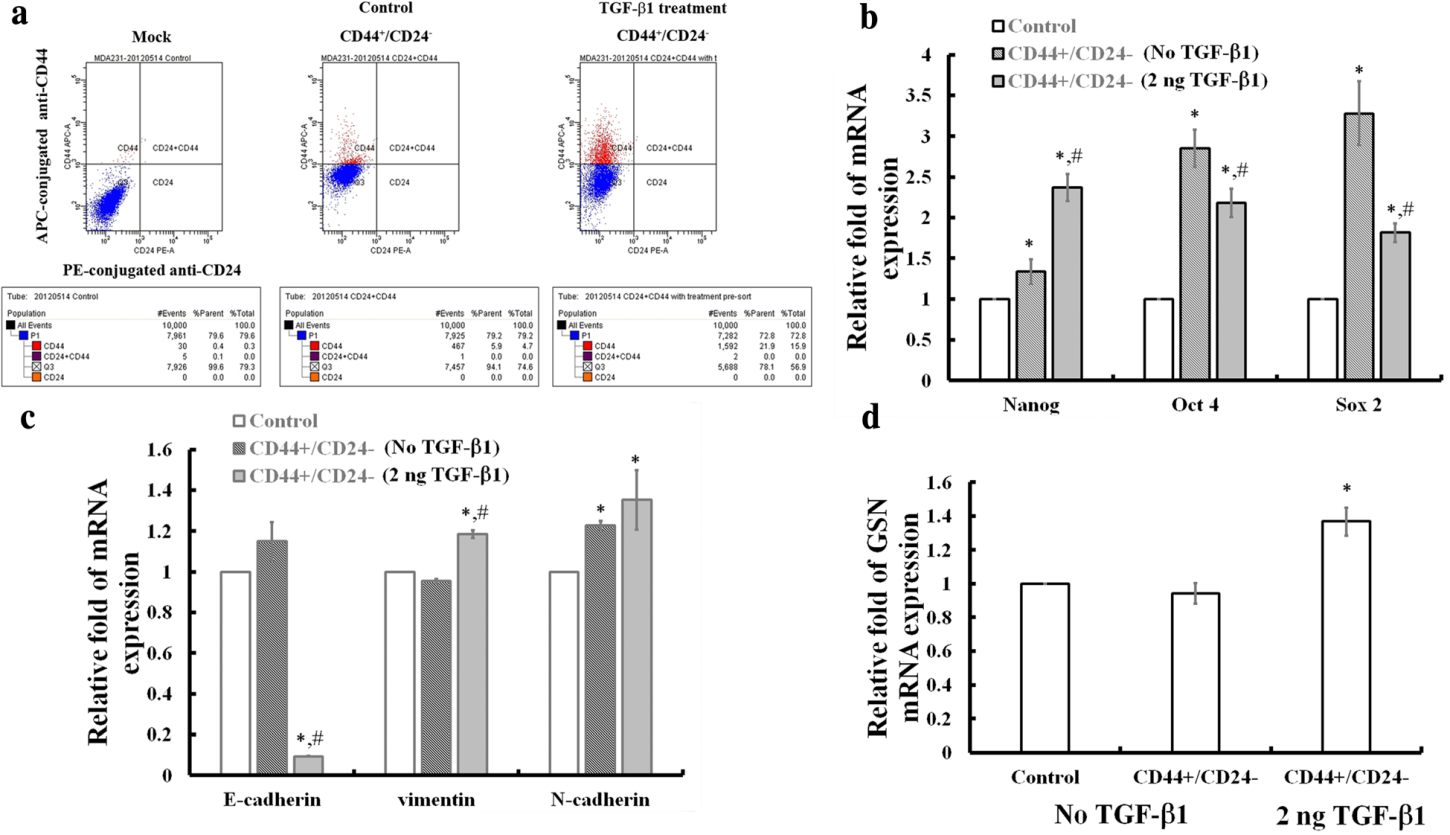
TGF-β1 induction increases the CD44+/CD24- subpopulation by coordinating gene expressions for CSC markers, EMT markers, and GSN in MDA-MB231 cells. **a** Fluorescence-activated cell sorting (FACS) analysis to measure CD44 and CD24 expression for MDA-MB231 cells with (right) or without (middle) additions of 2 ng/ml TGF-β1 for 3 days. CD44 and CD24 were detected by a combination of fluorochrome conjugated monoclonal antibodies against human CD44 (APC) and CD24 (PE), respectively. Mock (left) used as unstained control MDA-MB231 cells without TGF-β1. Data acquisition as shown below for each plot analysis. Real-time quantitative PCR (qPCR) analysis showing the increased gene expression for markers, including (**b**) stem cell pluripotency (i.e. Oct4, Sox2 and Nanog), (**c**) mesenchymal cell markers such as N-cadherin, vimentin, and E-cadherin, and for (**d**) GSN in post-sort CD44+/CD24- sub-population of MDA-MB231 cells with additions of 2 ng/ml TGF-β1 for 3 days. In **b, c**, and **d**, each value is the mean ± SEM (*n* = 6). *indicates significant difference compared to mock control, and # symbolizes a significant difference compared to post-sort CD44+/CD24- sub-population of MDA-MB231 cells without TGF-β1 treatment

To characterize CSC-like phenotypes in TGF-β1 treated cells, fluorescence-activated cell sorting (FACS) flow cytometry was used to isolate subpopulation of CD44+/CD24- for MDA-MB-231 cells. Under the condition of 2 ng/ml TGF-β1 for 3 days, the population of CD44+/CD24- MDA-MB231 breast cancer cells were increased (Fig. 5a). After collection by FACS flow cytometry, these cells were found to increase the gene markers for stem cell pluripotency (i.e. Oct4, Sox2 and Nanog) (Fig. 5b), the gene expression for mesenchymal cell markers such as N-cadherin, and vimentin, but to decrease in the gene expression for epithelial cell marker such as E-cadherin (Fig. 5c). This result indicated that TGF-β1 increases stem cell function and EMT in the CD44+/CD24- subpopulation of MDA-MB231 breast cancer cells. In addition, we also found that TGF-β1 increased the expression of GSN in the CD44+/CD24- MDA-MB231 cells (Fig. 5d).

### TGF-β1 induced epigenetic regulation of GSN gene expressions in the CD44+/CD24- subpopulation of MDA-MB231 cells

**Fig. 6:**
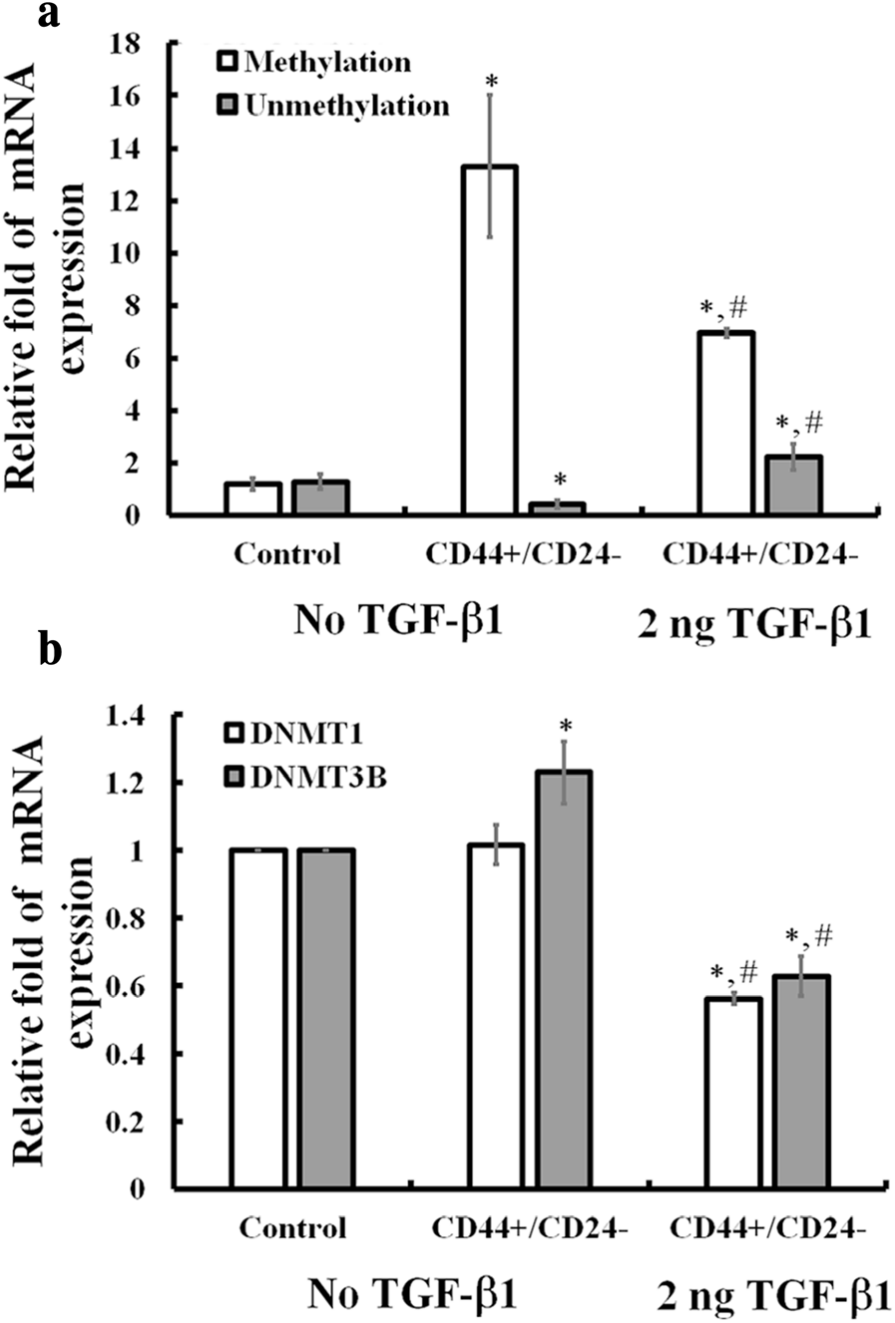
TGF-β1 induced epigenetic regulation of GSN gene expressions in the CD44+/CD24- subpopulation of MDA-MB231 cells. **a** Methylation specific PCR (MSPCR) analysis showed epigenetic regulation of GSN gene expression in the CD44+/CD24- subpopulation in MDA-MB-231 cells after TGF-β1 induction. **b** Real-time quantitative PCR analysis showed decreases of gene expression of two DNA methyltrasferase, DNMT1 and DNMT3B, in the CD44+/CD24- MDA-MB-231 cells subpopulation after TGF-β1 induction. The values are the means ± SEM (n = 6), with * indicating significant difference compared to mock control, and # symbolizing a significant difference compared to post-sort CD44+/CD24- sub-population of MDA-MB231 cells without TGF-β1 treatment

Alterations in GSN RNA expression in most breast cancers of rats, mice, and humans have been shown not due to gross mutations of the GSN gene [30]. Alternately, another route to modulate GSN expression is via epigenetic modification on GSN gene promotor [32–34]. To test whether the TGF-β1 causes the epigenetic modification on GSN expression in breast cancer cells, the method of methylation-specific PCR (MSPCR) for assessing the methylation and unmethylation on the CpG island at the promoter region of GSN (intron 1) was used in MDA-MB231 cells without FACS sorting (control), in CD44+/CD22- subpopulation sorted cells without TGF-β1 pretreatment, and in CD44+/CD22- subpopulation sorted cells with 2 ng/ml TGF-β1 pretreatment for 3 days (Fig. 6a). In control MDA-MB231 cells without TGF-β1 stimulation, CD44+/CD22- subpopulation sorting cells increased methylation by 10 folds but decreased unmethylation by 68 %. Interestingly, TGF-β1 pretreatment reduced 48 % methylation but increased 4 fold unmethylation of GSN promotor in the CD44+/CD22- subpopulation sorted cells (Fig. 6a). Consistently, two major DNA methyltransferases, DNMT 1 and DNMT 3B, were found to decrease their expression by 45 and 49 %, respectively, with TGF-β1 pretreatment in the CD44+/CD22 subpopulation sorted MDA-MB231 cells (Fig. 6b).

## Discussion

A subpopulation of cancer stem cells (CSCs) within heterogeneous metastatic breast tumors have the ability to differentiate into all the different cells found within a tumor and they have stem cell characteristics, including self-renewal, pluripotency, motility, tumor recurrence, and chemotherapy-resistance [35–37]. By sorting a subpopulation of CD44+/CD24-(low) cells from human breast cancer tissue, Al-Hajj et al. were the first to demonstrate that these cells can be enriched for breast CSCs and to develop a tumor in immune-deficient mice [18]. In addition, TGF-β has been shown to increase CSC numbers by producing gene markers linked to stem cell function and Epithelial to Mesenchymal Transition (EMT) in breast cancers [6]. More recently, evidence also showed that TGF-β could increase breast CSCs in the low claudin subtype of breast tumors [5]. In this study, we verified that with TGF-β1 treatment for 3 days the gene expression for CD44 and GSN was increased in MDA-MB231 cells (Fig. 1) with increasing the gene expression of EMT markers (Fig. 1) for enhancing their cell migration and invasion. Our study with FACS-flow cytometry also confirmed that TGF-β1 induction increased the CD44+/CD24- subpopulation of MDA-MB231 cells (Fig. 5a). In the TGF-β1 enriched CD44+/CD24- cells the mRNA expression levels for the markers of stem cell pluripotency (i.e. Oct4, Sox2 and Nanog) (Fig. 5b) were found to be increased in concomitance with the increased expression for mesenchymal cell markers (i.e. N-cadherin, Vimentin) but the decreased expression for epithelial cell marker (i.e. E-cadherin) (Fig. 5c). It is of note that the GSN expression level is higher for the TGF-β1 induction than for without TGF-β1 treatment (Fig. 5d) in the CD44+/CD24- subpopulation of MDA-MB231 cells. To test whether GSN plays a role in controlling cell proliferation and motility, we conducted GSN overexpression (GSN op) in the two human breast cancer cell lines of MDA-MB231 and MCF-7 (Figs. 2, 3). The results showed that GSN op altered cell morphology (Fig. 2) and increased cell surface elasticity with an increase in cell detachment, by which cause to increase the cell migration/invasion (Fig. 3). In addition, we also verified that GSN plays a role in the gene expression for the mesenchymal cell marker, vimentin in breast cancer cells with GSN op and/or siGSN approaches (Fig. 4). Taken together, the present study suggested that the modification of GSN expression might involve in the TGF-β1 signaling events for inducing cancer cell stemness and increasing cell migration and invasion in CD44+/CD24- subpopulation of breast cancer cells.

The regulation of GSN expression is varied in many different tumors [38–44]. In oral cancers, biphasic expression of GSN was found during the progression of carcinogenesis [40, 41]. Decreased GSN expression has been found in many transformed and malignant cancer cells, including breast cancers [42–44]. Evidence indicated that GSN gene loss is one of the most common disorders in invasive and metastatic breast cancers [45, 46]. Studies have shown that 71 % of human sporadic, invasive breast carcinomas and 56 % of ductal carcinomas in situ were strikingly deficient in the GSN protein [45, 46]. The clinical evidence also indicated that the GSN expression may be associated with survival from malignant breast cancers, and the frequency of GSN deficiency increases significantly with progression to invasive phenotypic cancer cells [45]. Recent studies have found the increased GSN expressions in chemo-resistant head and-neck (HNC) [47] and gynecological cancers [48]. These studies suggested that GSN might play important roles for chemoresistance in cancers. Interestingly, the present study showed that increased GSN expression is associated with the TGF-β1 signaling for breast CSC differentiation. Different cell populations of breast cancer cells vary their GSN expression in response to TGF-β1 induction (Fig. 5d). Only CSC-like cells (i.e. CD44+/CD24-) in breast cancer cells respond to TGF-β1 induction for increasing in GSN expression such as to maintain their invasive phenotype. It will be of interest to verify whether the TGF-β1 modified GSN expression is involved in chemoresistance in breast cancers.

Evidence showed that the GSN down-regulation is due to decreased activity of the GSN promoter by activating transcription factor-1 [49]. It was suggested that GSN expression and function can be further influenced by epigenetic changes [16]. Epigenetic modulation involves modifications of the transcriptional activation of certain genes [33, 34]. In the present study, we showed that the CpG island methylation of the GSN gene was decreased in CD44+/CD24- population of MDA-MB231 cells after TGF-β1 induction as compared to cells without treatment (Fig. 6a). Accordingly, TGF-β1 increased GSN gene expression in CD44+/CD24- population through decreases of DNA methylation of CpG island at GSN promotor by inhibition of two major DNA methyltransferase, DNMT1 and DNMT3B. These two DNA methyltransferase were decreased in the CD44+/CD24- subpopulation of MDA-MB231 cells after TGF-β1 treatment (Fig. 6b). Apparently, TGF-β1 induction attenuated the methylation but facilitated the unmethylation on GSN promoter region in breast cancer cells such as to remove the inhibition on GSN gene expression in MDA-MB231 cancer cells. Such information on the relationship between TGF-β1 and its control on DNA methyltransferase-dependent gene expression may have an important impact on the clinical therapy of patients with metastatic breast cancers.

## Conclusion

Our results suggested that TGF-β1 acting by epigenetic modulation of GSN gene expression might be linked to the signalling events for breast cancer stem cell differentiation.
